# Anthropophagy

**Published:** 1862-10

**Authors:** 


### Art. IX.—ANTHROPOPHAGY.

[There are in our possession materials for an extended, if not an exhaustive, investigation into necrophilism. We conceive that these observations take a wider range, and penetrate more profoundly into the nature of the instinct in which cannibalism, anthropophagy, and necrophilism, &c.—for these are different manifestations—originate; but it appears most prudent not to await the completion and maturing of these facts, but to introduce the following article from the pen of Dr. Le Grand du Saulle, as a suitable exordium to what is contemplated, although it neither represents the views of the translator nor embraces the scope of his inquiries.]

When we undertake the moral dissection of man, we must be startled at nothing; the unforeseen is a field without limits. It certainly shocks us to lay bare unsuspected sores; but are we to recoil from truth because it is ugly? The Athenian legislator did not provide for parricide. Everything has changed since the times of Solon, and justice is now often called upon to punish unnatural children. May not our descendants have to deplore some atrocity of which we have been neither witnesses nor accomplices? Let us, at all events, offer up our prayers that the isolated cases of anthropophagy which are collected and classified in this paper may remain rare examples of the most strange and the most horrible of all aberrations, and beyond the reach of imitation.

As a morbid condition, anthropophagy does not exist; as a crime, it is almost an impossibility; and we hope to prove that it should be regarded as a medico-legal monstrosity. It will not be superfluous to attempt an explanation of what may be the cause of so awful a psychical disorder. There are in the pathology of the soul two classes of functional disorders. I. In examining a lunatic, the physician detects extravagance in the conceptions, errors of judgment, limitation of intellectual combinations, incoherence, absurdity in the deportment, partial or total loss of memory, and exaltation or depression of the powers of the understanding. The patient forgets himself, is irrational, agitated, or depressed; he reels about in a circle of errors, takes his servant for a prince, his relations for enemies; he is miserable, criminal, lost, a king, emperor, or pope; he is nothing but an abject worm, or he calls himself Jesus Christ. II. The man of art, again, may observe a state of perversion of the propensities, of the natural sentiments, of the affections and passions; the will wanders without guide. Forgetfulness succeeds friendship, aversion takes the place of love, violence that of gentleness; the impulse to theft, murder, arson, suicide, deeply impresses a brain which knows not how to resist, and thus in certain cases, of extreme rarity, it is true, the alienation exceeds all experience, and corpses are exhumed and defiled by horrible caresses, or the habits of savages are imported amongst us, and human flesh serves as food. Dr. Barbaste, a learned member of the Medical Faculty of Montpellier, published, about five years ago, very curious researches as to anthropophagy, and this is almost the only scientific document extant upon this interesting question. More desirous of giving free scope to the habitual tendency of his imagination than to enter upon a practical medico-legal examination and discussion of facts which are so difficult to arrange, the author confined his observations within a very limited range, which we shall have an opportunity of enlarging, as the records of this exceptional criminality are, unfortunately, more abundant than he appears to have believed.

Anthropophagy was in past ages the result of prejudice and fanaticism. It was one of the terrible extremities to which men were reduced by famine; but since it has been introduced within the realm of pathology, it has been successively attributed to insanity, to chlorosis, or to a pretended organic tendency. These circumstances undoubtedly connect the subject with our art; but nevertheless, we believe that we shall be able to give a very summary exposition of the customs, institutions, or depraved instincts which have served as occasions or pretexts for anthropophagy. As is shown by M. Barbaste, the Lydians and Medes according to Herodotus, and the islanders in the Atlantic according to Plato, cemented their conspiracies by drinking human blood. Sallust attributes to the accomplices of Cataline, an act identically the same when he says—"They circulated human blood mixed with wine in cups." Tacitus speaks of the princes of Asia swearing allegiance upon their own blood, which they went so far as to drink. "Sanguis gustatus fœderibus." If we are to credit Juvenal, the Scythians quenched their thirst with the blood of their enemies, and the Tintirites devoured their flesh. The Gascons and the Sagontins formerly supported themselves on the flesh of their countrymen.

Without going back so far into history, have we not seen the Parisians devouring the bloody remains of the Marshal D'Ancre? The tyranny of hunger may compel man to descend even to the appetites of the carnivora. In describing the horrors which marked the siege of Rochelle, Anquetil relates that, urged by starvation, a father and mother dug up the scarcely cold body of their daughter, and ate it. We know, lastly, that the siege of Paris by Henry IV. was followed by most lugubrious consequences; that not only the horses, asses, cats, rats and mice were sacrificed as a very insufficient source of support, but they made meal from the dried bones collected in the cemeteries. "A mother," says a narrative of the period, "in imitation of what occurred during the siege of Jerusalem, roasted the limbs of her dead child, and died of grief under this revolting nourishment."

Having paid this tribute to history, let us strike the clinical balance of anthropophagy. "About the year 1600," says Professor Andral, "Jean Granier, a lad of fourteen, attacked with lycanthropy, traversed a district of which he was the terror. He repeatedly encountered young children and devoured them. He was arrested and brought before the courts of justice in Bordeaux, where these facts were established."* Gall has recorded an observation of an individual who, instigated by an irresistible propensity to eat human flesh, committed many assassinations in order to accomplish this object. The daughter of this person, although separated from her father, and educated at a distance from his family, yielded to the same craving. Prochaska quotes the case of a woman of Milan who attracted children to her home in order to slay them, and to salt and eat their flesh. The same author speaks likewise of a man who killed a traveller in order to devour him.†

Contemporary journals, and likewise certain scientific works, have recorded the misfortunes of a Scotch family, many members of which were hereditarily possessed by the imperious craving for human flesh. After recounting an instance of hereditary aversion to animal food, M. Lucas cites, as an illustration of the opposite tendency, a very ancient fact borrowed by Gaubius‡ from Hector Boetius. The latter mentions a girl, whose father had been instigated by so violent and irresistible a propensity to eat human flesh, as to lead him to commit murders; who, although separated from her father and mother, who were condemned to the stake before she was a year old, and although trained among virtuous guardians, yielded, like her father, to the inexplicable craving for human flesh. — *Traité Philosphique, &c., de l'Hérédité Naturelle,* T. i. p. 391.

Roderic a Castro speaks of a pregnant woman who strongly desired to eat the shoulder of a baker, and that she killed him. Languis tells of a female who longed, during gestation, to taste her husband's flesh—who killed him, and then salted the body in order to prolong the pleasure.

---

* *Pathologie Interne.* † *Opera Omnia,* T. ii. p. 98.

‡ Gaubius' account is very vague:—The father of the girl, a robber, "was said to have lived on man's flesh."—*Philosophical Discourse,* p. 100. (*Note by Translator.*)

In July, 1817, a workman left his home in order to beg in the neighbourhood. Upon his return, two hours afterwards, he asked his wife for his youngest child. "He is asleep," she replied, and pointed to a small closet. The father opened the door and discovers the body of his infant, without a leg. The unfortunate father goes out, and shortly after returns with the mayor. The accused, on being pressed, confessed without emotion, that in the extreme want by which she was surrounded she had killed her infant, had removed a leg, and cooked it with greens; that she had eaten a portion of this meal and preserved the remainder for her husband. They discovered a foot in a meat-safe, the remains of the vegetables, and a picked bone, which they recognised to be that of the child. It was found that, at the time of the murder, the mother was not destitute of provisions. The President of the Assize Court of Colmar was the first to institute an inquiry as to the integrity of the intellectual faculties in such cases. The polyphage, of whom M. Percy has transmitted a narrative, had the habit, among other and disgusting customs which are here omitted, of going into the slaughter-houses and waste places to dispute with dogs and wolves the most horrible carrion. The nurses of the hospital at Versailles, where he was placed, had detected him drinking the blood of such patients as had been bled, and in the dead-house sucking, like a modern vampire, that of the corpses.* The stomach of this man filled the whole of the abdominal cavity; and this exceptional condition has been assigned as a cause of his depraved instincts. We are not disposed to adopt this explanation.

On the 29th of November, 1824, Antoine Léger, aged twenty-nine, a vine-dresser and old soldier, was brought before the Assize Court of Versailles. The indictment informs us, that the accused always appeared sombre, stern, preferring solitude, and flying the society of females and young people of his own age. He left his father's house upon the 20th of June 1823; retreated to a wood, and after wandering about for a week discovered a cavern among the rocks. He installed himself in this retreat and subsisted for a month and a-half upon roots, pickles of wheat and berries. He, however, visited the village upon many occasions to buy victuals. He stole artichokes one night; and upon another he caught a rabbit, which he killed and devoured raw at a sitting.

"On the 10th of August," he says, "when I went to gather apples, I saw a little girl sitting at the edge of the wood. The idea seized me to carry her off. I passed my handkerchief round her neck and lifted her on my back; she cried slightly. I

* *R. d'Amador. La Vie du Sang*, note 7.

travelled through the wood and was hot, hungry, and thirsty. I remained for half-an hour unconscious; overcome by hunger I set about devouring her." Léger denied all that had been alleged as to rape and mutilation of the organs of generation; but confessed that, after opening the body, seeing the blood flow out in abundance, he quenched his thirst and sucked the heart of his victim before eating it. "I did all this," he said, "in order to get blood. I wished to drink blood; I was tormented by thirst; I was no longer myself."

The indictment imputes frightful callousness to Léger. They recounted to him all the circumstances of his crime: but "yes," uttered with indifference, was his only reply to every question addressed to him. During the examination it was remarked that his features presented an appearance of calm and gentleness; his expression was dull, his eyes fixed, his countenance motionless. He preserved the most profound impassibility, yet had an air of gaiety and self-satisfaction. Léger was condemned to death, and executed. "His head," says Georget, "was examined by Esquirol and Gall, in the presence of many other physicans. Esquirol observed many morbid adhesions between the brain and the pia mater."

Maria de las Dolores, inhabiting the mountains of Segovia, was seduced by Juan Diaz. Her lover, in order to save her honour, demanded her in marriage from her father, Pedro Dominguez, an old man of sixty-five. He angrily repulsed the claimant. From that time the shepherdess became melancholy and taciturn. She selected the most solitary spots for the pasture of her flocks, and held no communications with her companions. On the evening of the 20th March, 1826, she entered her hut after shutting up her sheep in the fold, and busied herself in roasting a morsel of meat. Her father was sleeping before the fire. Suddenly seized with a horrible frenzy, Dolores, armed with an andiron, knocks down her father by repeated blows. Her fury is redoubled by the sight of the blood; she throws herself upon her victim, opens the chest with a cutlass, drags out the still palpitating heart, places it beside the morsel of flesh upon the fire, and when it is half-roasted she begins to devour it. But forthwith she utters loud cries of despair, which resound far and wide. The shepherds rush from the adjoining cabins. What a frightful spectacle! By the side of the mutilated corpse there is a fury, who, with gory mouth and staring eyes, holds in her hand a piece of human flesh, which she shows to one of them, shrieking, "See, there is the heart of him who prevented me from being the happiest of women—of him who deprived me of the man whom I adore! It is the heart of my father, whom I have murdered! Taste it, if you like! It is the heart of my father!" The shepherds remained confounded—stupefied. Becoming more and more excited, Dolores set fire to her clothes, tore her bosom with her nails. She was arrested, and taken to Segovia; she had entirely lost her reason, and replied to the questions addressed to her by loud cries. The court of Segovia condemned her to seclusion in an asylum during her life.

M. Bertholled has published in the *Archives Générales* the case of a man whose favourite food consisted of the most disgusting animal substances, even of portions of dead bodies. He obtained access more than once to cemeteries, where, by the assistance of instruments, he exhumed bodies recently interred, in order greedily to devour the intestines. Finding in the abdomen sufficient to satisfy his appetite, he did not disturb other parts of the body. This individual, adds Dr. B., was about thirty years old, tall, and of a figure in no way indicating his revolting propensity. What was more extraordinary was, that he was not urged by an insatiable hunger. He did not eat ravenously, but filled his pockets with the remains of one meal until he was again inclined to eat. When interrogated as to the origin of his depraved appetite, his replies traced it back to his earliest infancy. He devoured a recently-interred corpse the same day he was arrested. This man might sooner or later have committed some outrage: he confessed that, although he never yet had attacked any living creature, he might, if pressed by hunger, have done so, had he found an infant asleep in his wanderings through the country. He was consigned to an asylum as a dement.*

On the 16th January, 1858, Jared and Clarissa Comstock, living near to the town of Hamilton, in the county of Madison, New York, were murdered They were about 70 years old, and held in general esteem. A neighbour, looking accidentally through the window, saw their lifeless bodies upon the floor. The man lay extended upon his back, "his left side presenting a gaping wound of about six inches in length: the heart had been removed." The woman lay in the same position a few feet distant; her left side had a gash of a similar kind, and the heart had likewise been dragged from the chest. The disorder and torn state of their dress showed that there had been a struggle. The two hearts were afterwards discovered in the oven of the stove, half roasted and half gnawed. Between the two corpses, and seated on a sofa, slept tranquilly William, the eldest son and murderer of the Comstocks. The police arrested the parricide, a man of 35 years of age, middle height, whose physiognomy indicated dulness rather than ferocity. William was regarded as gentle and inoffensive, and lived upon excellent terms with his

* *Archives Générales de Médecine,* T. vii. p. 472.

parents, who never complained of him. "My father still breathed when I tore out his heart, which I wanted. As to my mother the affair was easier, but my father was thicker skinned. I intended to have gone to my brother and sister, and finished the job, but I fell asleep." Labouring under epilepsy and hallucinations of the worst kind, William Comstock was unwilling to give any explanations as to the motive which impelled him to roast and eat a part of the heart of his aged parents. The jury, after declaring him to be insane, directed him to be confined in an asylum. We know that chlorotics are subject to unnatural appetites, and how frequently it happens that such patients yield to extravagant desires. We have read a fact in reference to this point in the *Courrier de la Drome*, of a girl of 14, who eagerly sought all opportunities of drinking human blood. She liked to suck that which ran from recent wounds. Lastly, as we have seen, females during pregnancy may commit similar acts.

Thus, as we intimated at the commencement of this article, anthropophagy is not entitled, any more than cretinism, to be included under mental pathology. The nosological arena has not been sufficiently widened as to give admission to these two anomalies, which it is convenient to class among *monstrosities*. Although, in the majority of cases, anthropophagy is connected with mental alienation, and proceeds most frequently from a psycho-cerebral neurosis, it is not less true that it deserves only the title of a horrible complication; that it consists in an act of all others the most repugnant to our manners, to morality, and to reason; and that its manifestations place the coping-stone upon the greatest of all misfortunes—the disturbance and annihilation of the intellectual powers. After the ruin of his understanding, the lunatic who indulges in anthropophagy, only obeys, as a machine, an impelling force which he cannot resist.

Society demands no retribution from him who at the moment of the crime has acted without judgment. All punishment which is necessarily ineffective is useless. If the fear of chastisement has not prevented, will punishment itself remedy the error?

In the case under consideration, how can we explain these outbreaks, which are so strongly in antagonism to the affections and passions of mankind? Would it not be a calumny upon humanity to suppose the spirit of that citizen healthy who is capable of devouring human flesh? The wretch bound to crime as the slave was bound to his chain, had he passed through every degree of immorality, could not reach so low as this hideous depravity.

The question of responsibility is resolved so far as anthropohagy is connected with a morbid state of the intellect. If, on the contrary, chlorosis, pregnancy, and perversion of the instincts are taken into account, we are not called upon to go in advance of the special rules provided ; all will depend upon the appreciation of the fact and of the concomitants of the fact. The more unnatural a crime is, the less necessary is it to seek its cause in ordinary motives. When the legal expert has taken the precaution, so wise in similar cases, of determining whether there has been simulation or not, he can only act according to the exigencies of the moment, and deliver, in all sincerity, that opinion which he believes to be most in harmony with the interests of science and truth. "The conception of the Just is one of the glories of human nature," says M. Cousin, and it is undoubtedly that conception which should guide our conscience. *Medici non proprie testes, sed est magis judicium quam testimonium.*

As to the jurists who demand that the criminal lunatic should be first treated medically, and then tried and condemned during one of those transient intermissions which characterize his disease, we have only one question to ask. Do they seriously think that it would contribute to the dignity of justice to watch for the glimmering light of an unsteady reason in order to make ready her sword ?—*Annales Medico-Psychologiques,* Juillet, 1862.

---

